# Machine learning models development for shear strength prediction of reinforced concrete beam: a comparative study

**DOI:** 10.1038/s41598-023-27613-4

**Published:** 2023-01-31

**Authors:** Zaher Mundher Yaseen

**Affiliations:** Civil and Environmental Engineering Department, King Fahd University of Petroleum and Minerals, Dhahran, 31261 Saudi Arabia

**Keywords:** Civil engineering, Applied mathematics, Computational science, Composites

## Abstract

Fiber reinforced polymer (FPR) bars have been widely used as a substitutional material of steel reinforcement in reinforced concrete elements in corrosion areas. Shear resistance of FRP reinforced concrete element can be affected by concrete properties and transverse FRP stirrups. Hence, studying the shear strength (*V*_*s*_) mechanism is one of the highly essential for pre-design procedure for reinforced concrete elements. This research examines the ability of three machine learning (ML) models called M5-Tree (M5), extreme learning machine (ELM), and random forest (RF) in predicting *V*_*s*_ of 112 shear tests of FRP reinforced concrete beam with transverse reinforcement. For generating the prediction matrix of the developed ML models, statistical correlation analysis was conducted to generate the suitable inputs models for *V*_*s*_ prediction. Statistical evaluation and graphical approaches were used to evaluate the efficiency of the proposed models. The results revealed that all the proposed models performed in general well for all the input combinations. However, ELM-M1 and M5-Tree-M5 models exhibited less accuracy performance in comparison with the other developed models. The study showed that the best prediction performance was revealed by M5 tree model using nine input parameters, with coefficient of determination (R^2^) and root mean square error (RMSE) equal to 0.9313 and 35.5083 KN, respectively. The comparison results also indicated that ELM and RF were performed significant results with a less slight performance than M5 model. The study outcome contributes to basic knowledge of investigating the impact of stirrups on *V*_*s*_ of FRP reinforced concrete beam with the potential of applying different computer aid models.

## Introduction

Fiber reinforced polymer (FPR) composites have been increasingly used in reinforcing concrete beams for flexural or shear strengthening^1,2^. These composites were used as a substitute of steel bars for reinforcing concrete structures in corrosive environment. In these circumstances, applying of FRP stirrups has more advantage than using longitudinal rebar because they are located as an outside bar with regard to flexural reinforcement^3^. FRP materials have been applied to prevent to the corrosion problem which is considered a serious issue in civil engineering structures^4,5^. FRP bars characterized by its ability to resist corrosion, light weight, high strength and good fatigue endurance^6^. However, they have some drawbacks such as low modulus of elasticity and linear elastic performance leads to failure, which indicated lower elastic behavior compared with steel reinforcement.

Shear strength (*V*_*s*_) of reinforced concrete beam is a result of several mechanisms like shear resistance of uncracked concrete, friction forces due to aggregate interlock, residual tensile resistance between inclined cracks and the *V*_*s*_ providing by dowel action and transverse bars^7,8^. Dowel action uses longitudinal bars to transfer the shear forces^9^. Aggregate interlock and cracked surfaces transfer shear friction of concrete. shear friction of concrete is influenced by the size of aggregate, crack size and the concrete strength^10^. High shear friction can be attained by increasing the size of crack and aggregate^10^. The depth of compression area and concrete strength also affected the *V*_*s*_. *V*_*s*_ decreases in concrete members has low concrete strength and shallow uncracked concrete area^10^. Residual tensile strength is a significant factor contributed to the shear forces in concrete members with small crack width^11^.

In FRP reinforced concrete, the mechanism is different. The mechanical characteristics of FRP bars affect the shear resistance result of traditional steel reinforcement beam. the contribution of compressed concrete to FRP reinforced concrete beam is different from traditional reinforced concrete beam^12^. The major difference is the neutral axis of FRP bar is lower than steel before reaching the yield point. FRP bar does not reach yield point which makes the compression area does not decrease while increasing the load up to rupture. Using of FRP bars in reinforced concrete beam leads to low shear stiffness, increase crack width, decrease friction forces and reduce the residual tension between inclined cracks. The experimental study by^13^, concluded that the *V*_*s*_ of longitudinal reinforcement of FRP bars is lower than steel reinforcement when using in concrete structure. The study by^12^, indicated that the influence of longitudinal bars on *V*_*s*_ can be neglected because it is lower than the influence of other mechanism.

The influence of transverse FRP bars determines by the value of stresses gained by the reinforcement. The value of stresses of FRP stirrups should be assessed because they do no reach yield point and they have linear elastic behavior up to rupture^14^. The early failure of FRP stirrups is happened when they are intercepting with the shear crack in the bent portion because this area is characterized by concentrated stress and the tensile strength of the bent bar is lower than the straight reinforcement^15,16^. After that, the stresses of the failed stirrups are moved to the other stirrups across the critical cracks leading to advanced failure of them. Hence, most design codes of FRP reinforcement determines the permitted value of strain in FRP stirrups at maximum point. According to this fact, the mechanism of *V*_*s*_ in the longitudinal and transverse FRP bars is the same as that in the traditional steel stirrups reinforced concrete beam. However, the *V*_*s*_ of concrete structure with FRP bars is less than that of structures using steel reinforcement stirrups due to low modulus of elasticity and developing of bigger and wider cracks leading to low shear resistance forces in structure components^17^. Several design codes and guidelines have developed shear design equations of FRP reinforced concrete beam including ACI-440.1R-06^18^, CNR-DT200/2003^19^, CSA S6-09 addendum^20^, CSA-S806-12^21^, JSCE^22^, ISIS-M03-01^23^. In these guidelines, the *V*_*s*_ of reinforced concrete members is calculated based on the influence of concrete and transverse FRP bars.

*V*_*s*_ mechanism is considered a complex process due to contribution of multiple parameters such as concrete and beam dimension parameters^24^. For predesign purposes, engineers are very much interested in determining the physical properties of FRP reinforced concrete beam. Over the past 2 decades, development of reliable model for *V*_*s*_ prediction is always an ambition for structural scholars^25–27^. Several studies have been conducted to propose empirical equations for *V*_*s*_ in concrete structures. Fico et al.^12^ reviewed the design guidelines and assess the current equations of shear prediction in FRP reinforcing member with and without stirrups. The study concluded that the minimum value of *V*_*s*_ was gained by^19^, with a coefficient of variation (COV) equal to 32% while JSCE^22^ was showed conservative results.

It is highly essential to exhibit some related researches to empirical formulations and codes design. Machial et al.^28^ compared the capacity of several models and guidelines such as CSA S6-09 addendum^20^, ISIS-M03-01^23^, the modified compression field theory^29^ and other models by using 46 samples with stirrups. The study showed that the best results have been gained by ISIS-M03-01^23^ with COV equal to 20.5%. The authors also concluded that ISIS-M03-01^23^ has produced unreliable outputs in calculating contribution of *V*_*s*_ while the best balance of accuracy and efficiency has attained by CSA S6-09 addendum^20^. Razaqpur and Spadea^30^ compared the predictive performance of the developed method for FRP shear prediction including CSA standard S806-12^21^, JSCE^22^, ACI-440.1R-06^18^, CNR-DT200/2003^19^, Hoult et al.^29^ by using 119 samples test. The results revealed that CSA standard S806-12 has attained the predictive accuracy with shear prediction value equal to 1.15 and COV of 20%. Marí et al.^31^ presented a conceptual predictive model for *V*_*s*_ prediction utilizing 1131 tests results of reinforced concrete beam with and without stirrups. The authors indicated that the presented model has obtained a good prediction performance through the value of COV. These empirical methods have showed some limitations such as they have different formulas, and they are always changing which lead to different results. Also, the developed methods have not the ability to apply to every shear test prediction. Therefore, there is always an enthusiasm to present a robust and reliable method for *V*_*s*_ prediction among concrete researchers.

In the recent years with the rapid development of soft computing algorithms, ML models have been effectively explored by concrete researchers^32–37^. However, the development of *V*_*s*_ prediction using ML models still need further exploration because the majority of studies have been focused on the contribution of concrete *V*_*s*_ without considering the influence of stirrups. Numerous studies have been conducted ML models on the concrete and structural engineering issues^38–40^. Jumaa and Yousif^41^ used three AI models called artificial neural network (ANN), gene expression programming (GEP) and nonlinear regression to predict shear capacity of FRP reinforced concrete elements. The study showed that the developed models exhibited an excellent performance as compared with other models Development of generalized regression neural network (GRNN) was conducted to predict shear capacity of FRP reinforced concrete members without stirrups^42^. The developed model was compared with the design codes like ACI 440.1R, CSA S806 and JSCE. The results proved that GRNN had more accurate results that existing design codes. Chou et al.^43^ integrated a smart firefly algorithm (SFA) with least squares support vector regression (LSSVR) to predict *V*_*s*_ using different types of reinforced concrete beam including dataset with and without stirrups and with FRP reinforcement. Based on the comparison assessment with different ML models and empirical formulations, the developed model showed an outperformance accuracy than the others in *V*_*s*_ prediction. Abuodeh et al.^44^ employed a resilient back propagation neural network (RBPNN) and recursive feature selection elimination (RFE) to predict shear capacity of reinforced concrete beam strengthened with FRP laminates. The study revealed that the presented model attained accurate results than that using RBPNN with feature selector algorithm.

Alam et al.^45^ investigated the capacity of shear capacity prediction of FRP reinforced concrete members without stirrups by hybridize support vector regression (SVR) and Bayesian optimization algorithm (BOA). The results revealed that the developed model has more robustness than the classical SVR model and empirical equations. Nikoo et al.^46^ integrated bat algorithm with ANN to estimate shear behavior of FRB reinforced concrete elements. Based on the statistical assessment and comparison with other optimization algorithms, the study confirmed that the integrated model attained more accurate results than particle swarm optimization (PSO) and genetic algorithm (GA). Ebid and Deifalla^47^ used genetic programming (GP) to predict the capacity of FRP reinforced concrete beam with and no stirrups. The results revealed that utilized method gained more accurate results as comparing with that used in literatures. Alam et al.^48^ presented a hybrid ML called ANN-BOA for *V*_*s*_ prediction of reinforced concrete elements with FRP reinforcement without stirrups. The study indicated that the presented model showed better results than traditional ANN and empirical equations. Nguyen and Nguyen^49^ estimated *V*_*s*_ of FRP reinforced concrete beams without stirrups by training ANN model with four algorithm named Levenberg–Marquardt (ANN-LM), Quasi-Newton (ANN-QN), Conjugate gradient (ANN-CG) and Gradient Descent (ANN-GD). The outcomes of statistical measurement showed revealed the reliability and efficiency of ANN model in *V*_*s*_ prediction. Other studies presented tree base models of *V*_*s*_ prediction like random forest^50,51^, XGBoost^52,53^ and M5 model^54^.

Based on the motivation of integration of using the influence of transverse reinforcement in shear reinforcement models and soft computing methods, this work aims to develop advance ML models to simulate *V*_*s*_ capacity of FRP reinforced concrete beam using longitudinal and transverse stirrups. Three ML models were proposed in this paper including M5tree, random forest (RF) and extreme learning machine (ELM) to estimate the shear behavior based on collected dataset form previews literature studies. Different inputs were build based on statistical correlation and Their impact was explored by using the developed models. The first contribution of this study is quantifying the *V*_*s*_ of FRP reinforced concrete beam with transverse reinforcement, which has explored in limited studies. Secondly, advance ML models with different input combinations were developed to imitate shear behavior of reinforced concrete beam. Finally, this study provides the structural engineers with a reliable model have the ability to solve complex problems and predict shear behavior with an accurate predictive performance.

## Dataset description

To propose ML model, 112 samples of FRP reinforce concrete beams with FRP transverse reinforcement that failed in shear behavior have been collected from different previous studies^13,55–68^. The dataset include beam width (b), effective depth (d), concrete compressive strength (${{f}^{^{\prime}}}_{c}$), reinforcement ratio ($\rho$), modulus of elasticity for longitudinal reinforcement (${E}_{r}$), reinforcement ratio of transverse stirrups (${\rho }_{t}$), modulus of elasticity for transverse stirrups (${E}_{t}$), tensile strength of transverse stirrups (${f}_{u.t}$), ratio between shear span and effective depth (a/d) and shear strength of FRP reinforced beam (*V*_*s*_). The statistical properties showed that the maximum and minimum value of *V*_*test*_ are 20.5 and 590, respectively. They also indicated that ${{f}^{^{\prime}}}_{c}$ and b indicated high kurtosis with values more than 3. The statistical properties of the dataset are presented in Table 1.

**Table 1:** Statistical characteristics of collected samples.

	b (mm)	d (mm)	$${{f}^{^{\prime}}}_{c}$$ (MPa)	$$\rho $$ (%)	$${E}_{r}$$ (GPa)	$${\rho }_{t}$$ (%)	$${E}_{t}$$ (GPa)	$${f}_{u.t} $$ (MPa)	a/d	*V*_*s*_ (KN)
Mean	218.616	297.1696	36.5732	1.5421	73.6785	0.5214	73.2767	1056.0714	2.5937	162.6821
Standard error	6.5306	11.7986	1.06094451	0.0695	3.03908	0.0427	3.2106	34.8590	0.0709	9.4593
median	200	253	34.95	1.61	58	0.35	59	1100	2.5	130
Mode	250	253	39.4	1.89	56	0.12	94	1284	1.8	83
Standard deviation	69.1139	124.8648	11.2279	0.7357	32.1626	0.4527	33.9788	368.9137	0.7513	100.1082
Sample variance	4776.7431	15,591.2412	126.0675	0.5413	1034.4362	0.2049	1154.5623	136,097.3462	0.5645	10,021.6571
Range	307	767	64.2	3.14	111	1.46	114	1718	3.1	569.5
Minimum	150	170	20	0.51	29	0.04	30	322	1.2	20.5
Maximum	457	937	84.2	3.65	140	1.5	144	2040	4.3	590
Sum	24,485	33,283	4096.2	172.72	8252	58.4	8207	118,280	290.5	18,220.4
Count	112	112	112	112	112	112	112	112	112	112

## Methods overview

### Extreme learning machine (ELM)

**Figure 1:**
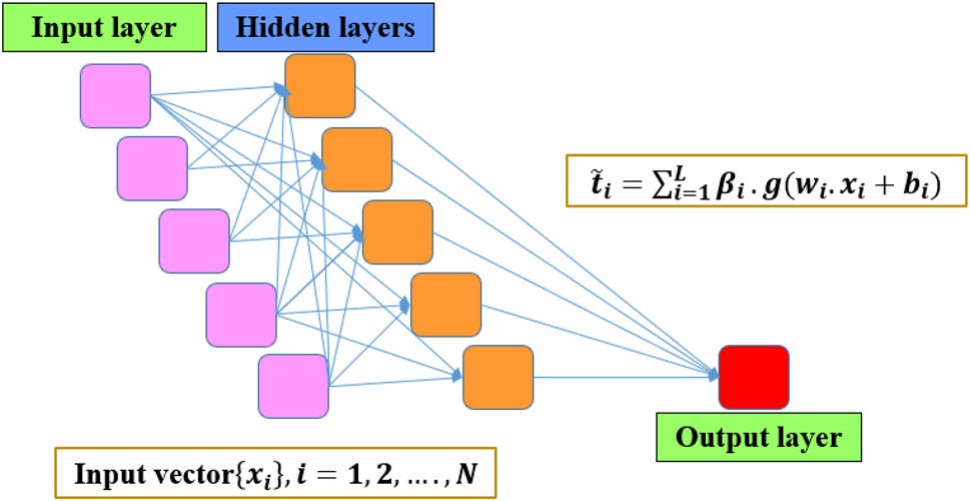
Structure view of ELM algorithm.

Extreme learning machine (ELM) is a new advance machine learning algorithm has been developed recently by Huang et al.^69^. The aim of proposing ELM is to enhance the performance of traditional single layer feed forward neural network. The significant step of ELM processing in the initialization of random hidden neuron and using Moore–Penrose generalized inverse method to determine the output weights of algorithm^70,71^. The learning algorithm during training phase of traditional neural network tunes the network parameters in iterative manner. In ELM method the process is different, the algorithm assumes the weight of hidden neurons randomly and the output weight is calculating using the least square method^72^. According to this, the weights of hidden neuron are remaining the same and the iterative loop is not needed. The hidden neurons of ELM algorithm create a random feature map to perform a nonlinear network between input parameters^73^. In random feature map, input parameters are separating linearly using the nonlinear network and this mechanism simplifies the training process of ELM. ELM characterized by its quickly learning phase and excellent generalized results^74^. ELM network combines from three layers including input, hidden and output layer. The paradigm of ELM algorithm is illustrated in Fig. 1.

A conventional feed forward neural network with L hidden neuron and *g*(*x*) activation function can be stated as below:

1 $${f}_{L}\left({x}_{j}\right)=\sum_{i=1}^{L}{\beta }_{i}.g\left({w}_{i}.{x}_{i}+{b}_{i}\right) \quad j=\mathrm{1,2},\dots .,N$$

${\beta }_{i}$ represents the weight vector and *N* is the number of training data. The output weight of hidden layer can be defined by a symbol *H* and the above formula can be reconstructed as follow:

2 $$H\beta =T$$

The first step of ELM construction is assuming input bias ${b}_{i}$ and hidden weights ${w}_{i}$. Secondly calculating *H* and finally determining the output matrix as below:

3 $$\widetilde{\beta }={H}^{ \dag  }T$$

${H}^{ \dag  }$ represents the inverse of *H* and refers to Moore–Penrose generalized inverse method in ELM model. *T* presents the outcome of the learning process of the regression formula. The ELM model training functions were; *nhid* was 1000, *actfun* was *purelin*, *init_weights* was *uniform_negative*, bias and verbose was set as true. Dataset was treated as matrix.

### Random forest (RF)

Random forest (RF) is a tree based model has been introduced by^75,76^ as an improvement of bagging tree method. RF is an ensemble tree method that builds a number of decision tree based on bootstrap sampling method performed through training phase. Single decision tree contains three components including the internal node, branch and the leaf node. Internal node denotes to an assessment of prediction problem. The output of this assessment is presented by the branch node, where the leaf node represents the class label of regression. In branch node, the mean of data points and mean squared error between these data were computed. This process is continuing until the mean squared error of regression tree reached the optimal value, then decision tree stops the growing process.

**Figure 2:**
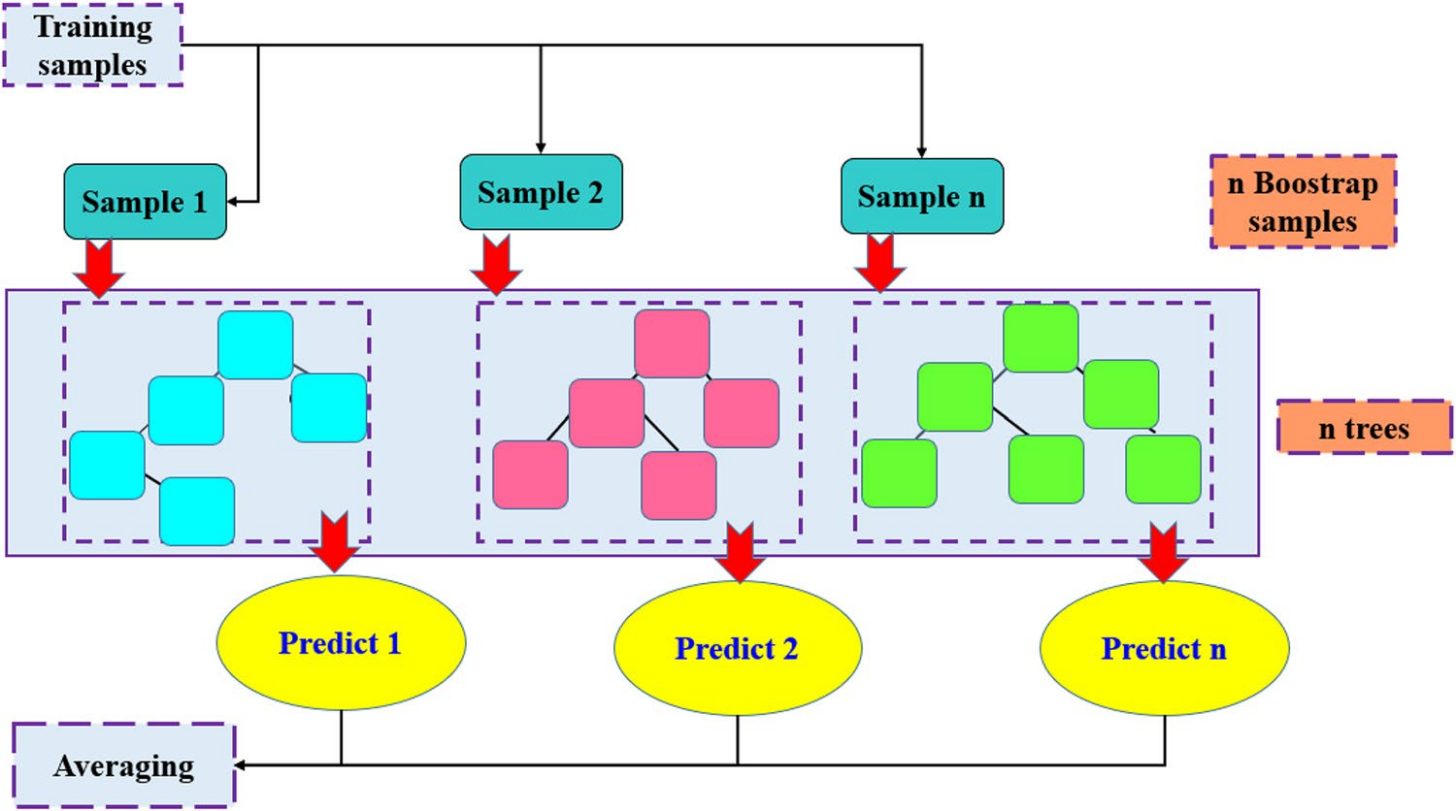
Graphical structure of RF model.

The construction process of RF model including the following steps: at first the training data set divided into two parts of data. The first part equal to two third of training sample and the second named the bootstrap sample which is equal to one third of original data set. The second step including modelling of RF algorithm by constructing a regression tree for each bootstrap sample created during training process. According to this step, a number of regression trees were generated, and the optimal attributes were selected based on random selection of max depth attributes for each branch node. after numbers of training cycles, the sequence of the developed regression tree is reached, which is considered in developing process of RF model. The final step of RF modelling is collecting the prediction results of decision tree and using the average formula to calculate the outcome of the new predictor. The mathematical expression of RF model is shown below:

4 $${\widehat{f}}_{rf}^{N}(x)=\frac{1}{N}\sum_{N=1}^{N} {t}_{i}(x)$$

where ${\widehat{f}}_{rf}^{N}(x)$ represents the incorporated regression tree, *N* the number of regression algorithm and ${t}_{i}(x)$ is the individual regression tree algorithm. The graphical presentation of RF method is depicted in Fig. 2. For the RF model development, *trainControl* function for *cv* method was determined as 5; *expand.grid* function for *mtry* was set between 1 to 20.

### M5 tree model

**Figure 3:**
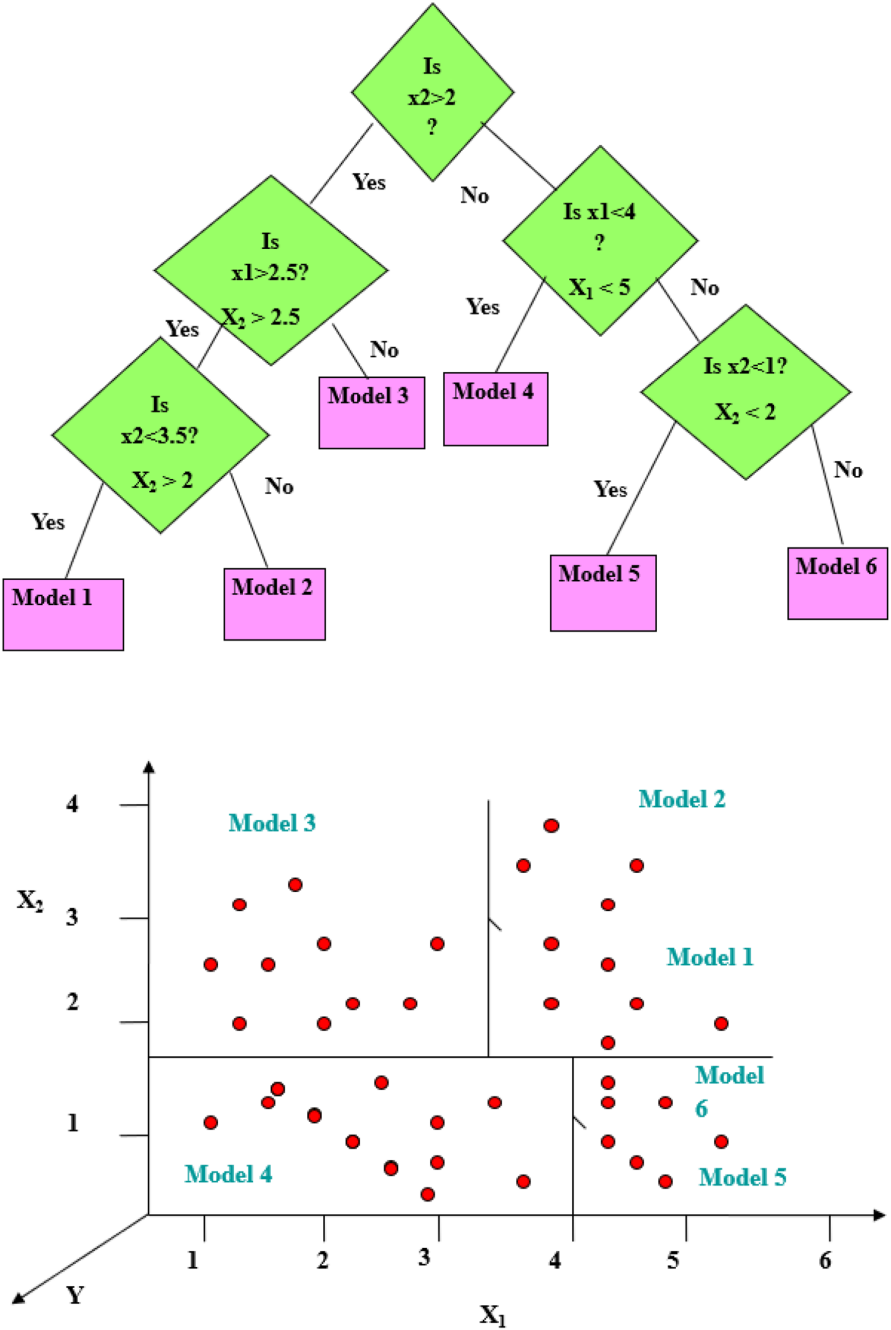
Schematic structure of M5 tree model.

M5 tree algorithm was developed by Quinlan^77^ to enhance the predictive performance of classical regression tree. The algorithm divides the training data set into subsets and develops a multiple linear regression model for each set of data. The main merit of M5 algorithm is handling complex and high dimension data with small size as compared with classical regression tree^78,79^. It has the same structure of regression tree and its construction based on dividing the samples at training process. The major difference from traditional algorithm is using of linear function at leave node as an alternative of discrete class label. Using of linear function in M5 algorithm instead of discrete label enables the model to handle continues numerical values and generalizes its application in regression problem. Another difference is the selection process of attributes values; the algorithm selects the attribute value that reduce the variability between classes instead of using information metric. The variation between values at each node is computing by measuring the standard deviation of attributes and calculating the reduced error that results from examining attribute values at the same node. The attribute is selected by algorithm if he attained the less error and this process is continued until the variation between values at each node reaches the minimum point^80,81^. Standard error reduction (SDR) between attributes can be calculated using the mathematical formula as below:

5 $$SDR=sd\left(T\right)-\frac{\left|{T}_{i}\right|}{\left|T\right|} sd({T}_{i})$$

where $sd$ mean the standard deviation, T is the set of attribute at each node and ${T}_{i}$ represents the output of that attribute. The output model for the subset division can be expressed by $O={a}_{0}+{a}_{1}{x}_{1}+{a}_{2}{x}_{2}+\cdots ,$ where $a$ represents the coefficient of linear function, $x$ is the input parameter and $O$ is the output value. The schematic structure of M5 tree model is presented in Fig. 3, which illustrated the process of division into subsets and development of linear regression model for input parameters. The M5 model was trained using *trControl* method for none; *expand.grid* function for pruned was set No, smoothed was set Yes, rules were set No, *preProc* was set for both center and scale. The dataset was treated as vector values.

### Modelling process and statistical assessment

The ability of ML models in predicting *V*_*s*_ behavior of FRP reinforced concrete beam with stirrups is examined by developing three algorithms named ELM, RF and M5. The algorithms constructed based on several parameter combinations by computed the correlation relationship between input and target parameter. Correlation values and input construction are reported in Tables 2 and 3.

**Table 2:** correlation values between predictors and FRP reinforced concrete *V*_*s*_.

Parameters	${V}_{test}$
b (mm)	0.76062
d (mm)	0.48338
${{f}^{^{\prime}}}_{c}$ (MPa)	0.02290
$\rho$ %	0.37630
${E}_{r}$ (GPa)	− 0.18682
${\rho }_{t}$ (%)	0.59767
${E}_{t}$ (GPa)	0.06225
${f}_{u.t}$ (MPa)	− 0.25977
a/d	− 0.47397

**Table 3:** Parameter combinations for the developed ML models.

Models	Parameter combinations
M1	${V}_{test}=b$
M2	${V}_{test}=b,{\rho }_{t}$
M3	${V}_{test}=b,{\rho }_{t},d$
M4	${V}_{test}=b,{\rho }_{t},d,a/d$
M5	${V}_{test}=b,{\rho }_{t},d,a/d,\rho$
M6	${V}_{test}=b,{\rho }_{t},d,a/d,\rho ,{f}_{u.t}$
M7	${V}_{test}=b,{\rho }_{t},d,a/d,\rho ,{f}_{u.t},{E}_{r}$
M8	${V}_{test}=b,{\rho }_{t},a/d,\rho ,{f}_{u.t},{E}_{r},{E}_{t}$
M9	${V}_{test}=b,{\rho }_{t},a/d,\rho ,{f}_{u.t},{E}_{r},{E}_{t},{{f}^{^{\prime}}}_{c}$

Based on the reported correlation values and parameter combinations, it can be noted that the first model includes the beam width (*b*) which has a good correlation with *V*_*s*_. The second input combinations include beam width (*b*) and reinforcement ratio of transverse stirrups (${\rho }_{t}$) as they have the highest correlation value with *V*_*s*_. Effective depth (*d*) was the third correlated parameters which added to the third model in addition to *b* and ${\rho }_{t}$ parameters. Parameters a/d, ${f}_{u.t}$ and ${E}_{r}$ have a negative correlation with *V*_*s*_. The least correlation was attained by the parameters ${E}_{t}$ and ${{f}^{^{\prime}}}_{c}$ where they included in model eight and nine. Several performance evaluators including coefficient of determination (R^2^), root mean square error (RMSE), mean absolute error (MAE), mean absolute percentage error (MAPE), Nash–Sutcliffe efficiency (Nash) and agreement index (MD) were conducted to validate the performance of ML models^82,83^.

6 $${R}^{2}={\left(\frac{\sum_{i=1}^{N}\left({V}_{sp}-\overline{{V }_{sp}}\right).\left({V}_{so}-\overline{{V }_{so}}\right)}{\sqrt{\sum_{i=1}^{N}{\left({V}_{sp}-\overline{{V }_{sp}}\right)}^{2}.\sum_{i=1}^{N}{\left({V}_{so}-\overline{{V }_{so}}\right)}^{2}}}\right)}^{2}$$

7 $$RMSE=\sqrt{\frac{\sum_{i=1}^{N}{({V}_{sp}-{V}_{so})}^{2}}{N}}$$

8 $$MAE=\frac{\sum_{i=1}^{N}\left|{V}_{sp}-{V}_{so}\right|}{N}$$

9 $$MAPE=\frac{1}{n}\sum_{i=1}^{N}\frac{\left|{V}_{sp}-{V}_{so}\right|}{{V}_{sp}}$$

10 $$\mathrm{NSE}=1-\frac{\sum_{i=1}^{N} {\left({V}_{sp}-{V}_{so}\right)}^{2}}{\sum_{i=1}^{N} {\left({V}_{so}-\overline{{V }_{so}}\right)}^{2}}$$

11 $$\mathrm{MD}=1-\frac{\sum_{i=1}^{N} {\left({V}_{so}-{V}_{sp}\right)}^{j}}{\sum_{i=1}^{N} {\left(\left|{V}_{sp}-\overline{{V }_{so}}\right|+\left|{V}_{so}-\overline{{V }_{so}}\right|\right)}^{j}}$$

where ${V}_{so}$ and ${V}_{sp}$ represents the observed and predicted parameters of shear strength; $\overline{{V }_{so}}$, $\overline{{V }_{sp}}$ are the average amount of the observed and predicted parameters of shear strength; N is the number of simulated samples.

## Application results and discussion

### Statistical evaluation

In the current work three ML models were applied to simulate *V*_*s*_ of FRP reinforced concrete with transverse reinforcement. Combinations of different input parameters were adopted to explore the ability of the developed models in *V*_*s*_ prediction. Tables 4 and 5 stated the statistical validation for training and testing data, respectively. The tabulated results indicated that M5 and RF models demonstrated a superior prediction performance with few predictors over the training phase with coefficient of determination equal to 0.70635 and 0.72679 for M5 and RF models, respectively. ELM model has achieved less prediction accuracy with few input parameters in comparison with the other models over training phase with coefficient of determination equal to 0.45874 using one input parameter. The best statistical performance for training data was attained using RF model with R^2^ = 0.96093, RMSE = 16.1986, MAE = 11.5136, MAPE = 0.07407, Nash = 0.95751 and MD = 0.91196. RF and M5 models exhibited an excellent prediction accuracy with one and two parameters over testing phase while ELM achieved less statistical performance using one predictor and its accuracy was enhanced by using more predictors. Among all models, M5 model gained the best predication accuracy with R^2^ = 0.9313, RMSE = 35.5083, MAE = 30.9291, MAPE = 0.51409, Nash = 0.89363 and MD = 0.83623.

**Table 4:** Statistical performance validation for training phase.

	R^2^	RMSE	MAE	MAPE	Nash	MD
**M5 model**						
M1	0.70635	46.5984	34.9096	0.26724	0.64838	0.6911
M2	0.7256	42.4761	32.9962	0.24113	0.70784	0.72222
M3	0.7544	39.6637	30.2293	0.21074	0.74525	0.75286
M4	0.80566	36.8915	27.5701	0.1916	0.77962	0.77026
M5	0.60868	55.2009	40.4591	0.25133	0.50658	0.61218
M6	0.8579	35.1328	26.3841	0.18697	0.80013	0.76983
M7	0.83087	37.3329	28.9575	0.21361	0.77431	0.74057
M8	0.82071	36.4512	27.6423	0.20512	0.78484	0.75931
M9	0.85785	33.3423	23.7891	0.17283	0.81998	0.79661
**ELM model**						
M1	0.45874	57.8147	43.2083	0.33506	0.45874	0.59854
M2	0.67977	44.4701	32.8803	0.22901	0.67977	0.73491
M3	0.68227	44.2962	33.0725	0.22914	0.68227	0.73231
M4	0.70819	42.4507	31.6697	0.21577	0.70819	0.74884
M5	0.74325	39.819	29.7108	0.21078	0.74325	0.76874
M6	0.74409	39.7538	29.7233	0.21057	0.74409	0.7688
M7	0.81169	34.101	26.3768	0.17699	0.81169	0.79586
M8	0.82235	33.1223	25.9927	0.1772	0.82235	0.79922
M9	0.85318	30.1113	22.6016	0.15456	0.85318	0.82798
**RF model**						
M1	0.72679	41.0759	31.9846	0.23532	0.72679	0.75449
M2	0.8381	31.9949	24.0258	0.1675	0.83424	0.813
M3	0.83394	32.4423	24.1769	0.16896	0.82957	0.81232
M4	0.90586	24.7125	18.8525	0.13166	0.90111	0.85439
M5	0.9298	21.0954	15.6778	0.09606	0.92794	0.88005
M6	0.9371	20.074	15.0502	0.09372	0.93475	0.88469
M7	0.93513	20.2367	14.8808	0.0889	0.93369	0.8867
M8	0.93635	20.3188	15.1434	0.09305	0.93315	0.8839
M9	0.96093	16.1986	11.5136	0.07407	0.95751	0.91196

**Table 5:** Statistical performance validation for testing phase.

	R^2^	RMSE	MAE	MAPE	Nash	MD
**M5 model**						
M1	0.87278	66.1925	58.4487	0.96696	0.63037	0.60884
M2	0.91138	57.4213	49.4181	0.96678	0.72184	0.69222
M3	0.90497	49.1965	43.2752	0.75717	0.79582	0.74345
M4	0.89804	46.7961	40.5897	0.51375	0.81526	0.75979
M5	0.64839	74.5231	63.2068	0.64038	0.53148	0.57852
M6	0.90307	45.0921	38.8302	0.58847	0.82847	0.77286
M7	0.91821	43.7888	39.1611	0.60512	0.83824	0.77152
M8	0.92017	39.8316	35.7796	0.58265	0.86615	0.80028
M9	0.9313	35.5083	30.9291	0.51409	0.89363	0.83623
**ELM model**						
M1	0.53875	76.5175	68.3818	0.90183	0.50606	0.53768
M2	0.87964	48.9455	43.9115	0.75206	0.7979	0.74537
M3	0.85948	49.0335	43.8525	0.71248	0.79717	0.74762
M4	0.8875	37.7118	29.4719	0.30186	0.88002	0.84523
M5	0.8926	36.1731	28.3248	0.2276	0.88961	0.85102
M6	0.89374	36.0325	27.7879	0.20975	0.89047	0.85383
M7	0.92212	34.4591	28.998	0.44497	0.89983	0.86144
M8	0.92614	33.832	27.9347	0.44842	0.90344	0.8695
M9	0.91524	42.5785	35.0837	0.69063	0.84706	0.84142
**RF model**						
M1	0.87907	54.2559	46.7718	0.78878	0.75166	0.71589
M2	0.90838	55.615	46.9295	0.90997	0.73906	0.70955
M3	0.90494	52.6812	43.8714	0.82987	0.76587	0.73418
M4	0.91726	42.7	36.3169	0.68527	0.84618	0.79463
M5	0.87551	47.025	40.2248	0.77561	0.81344	0.77431
M6	0.86674	48.2566	41.7767	0.77975	0.80354	0.75862
M7	0.8831	44.9501	39.349	0.70681	0.82954	0.77594
M8	0.90375	47.135	39.3565	0.74043	0.81257	0.76814
M9	0.89683	45.5893	39.4276	0.7795	0.82466	0.7801

### Graphical evaluation

**Figure 4:**
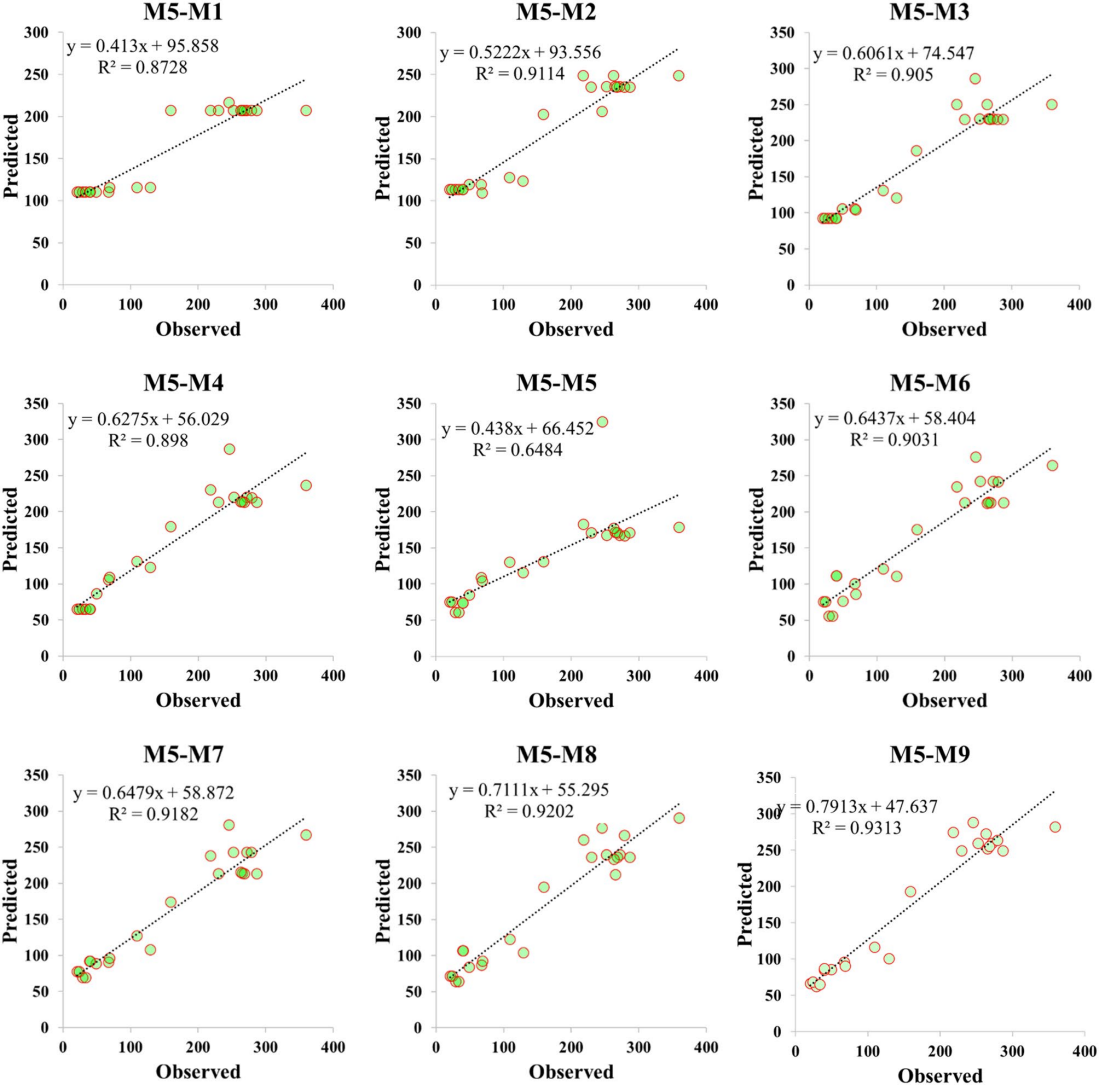
Scatter plot presentation of M5 prediction model over the testing modeling phase.

**Figure 5:**
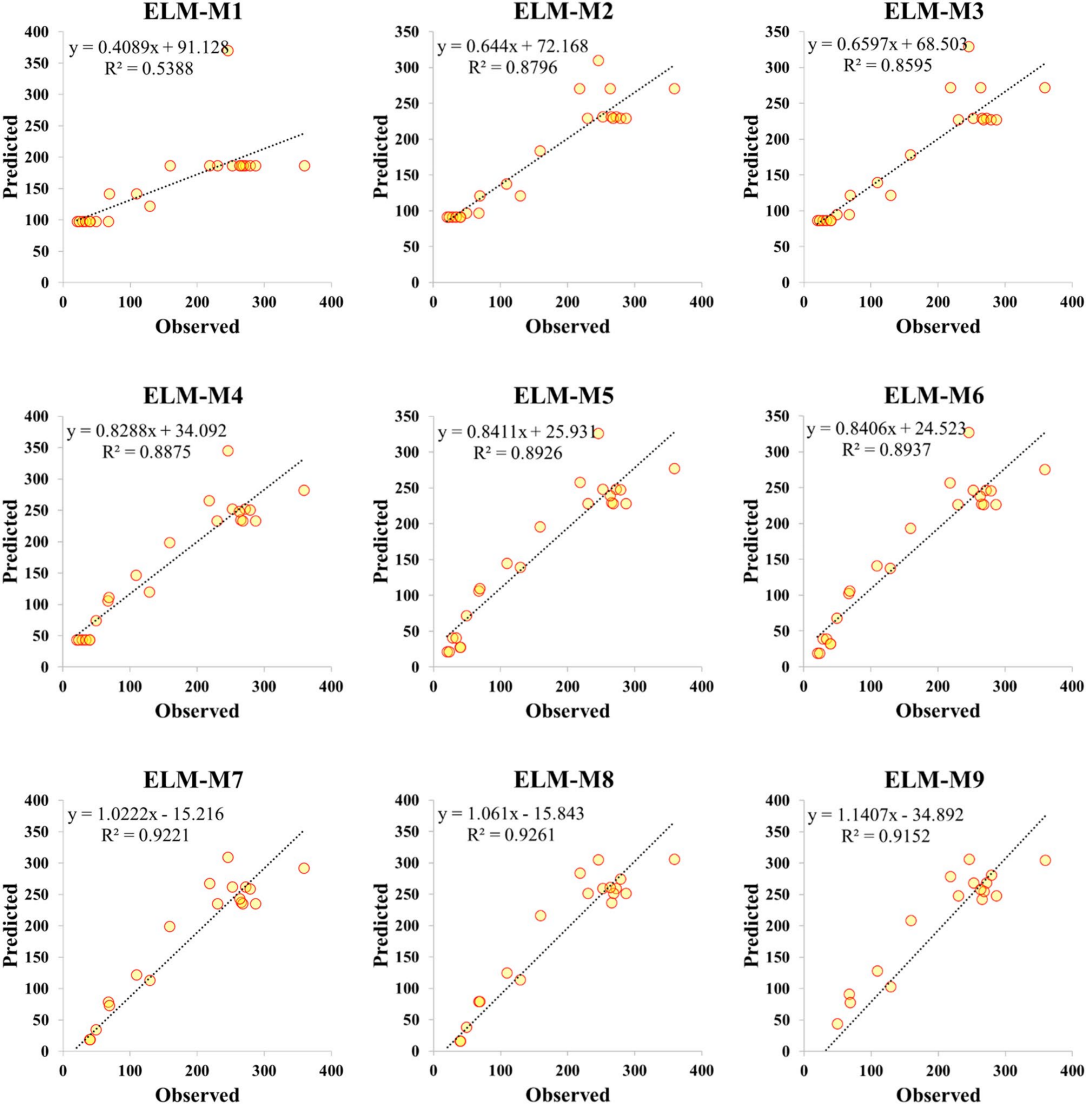
Scatter plot presentation of ELM prediction model over the testing modeling phase.

**Figure 6:**
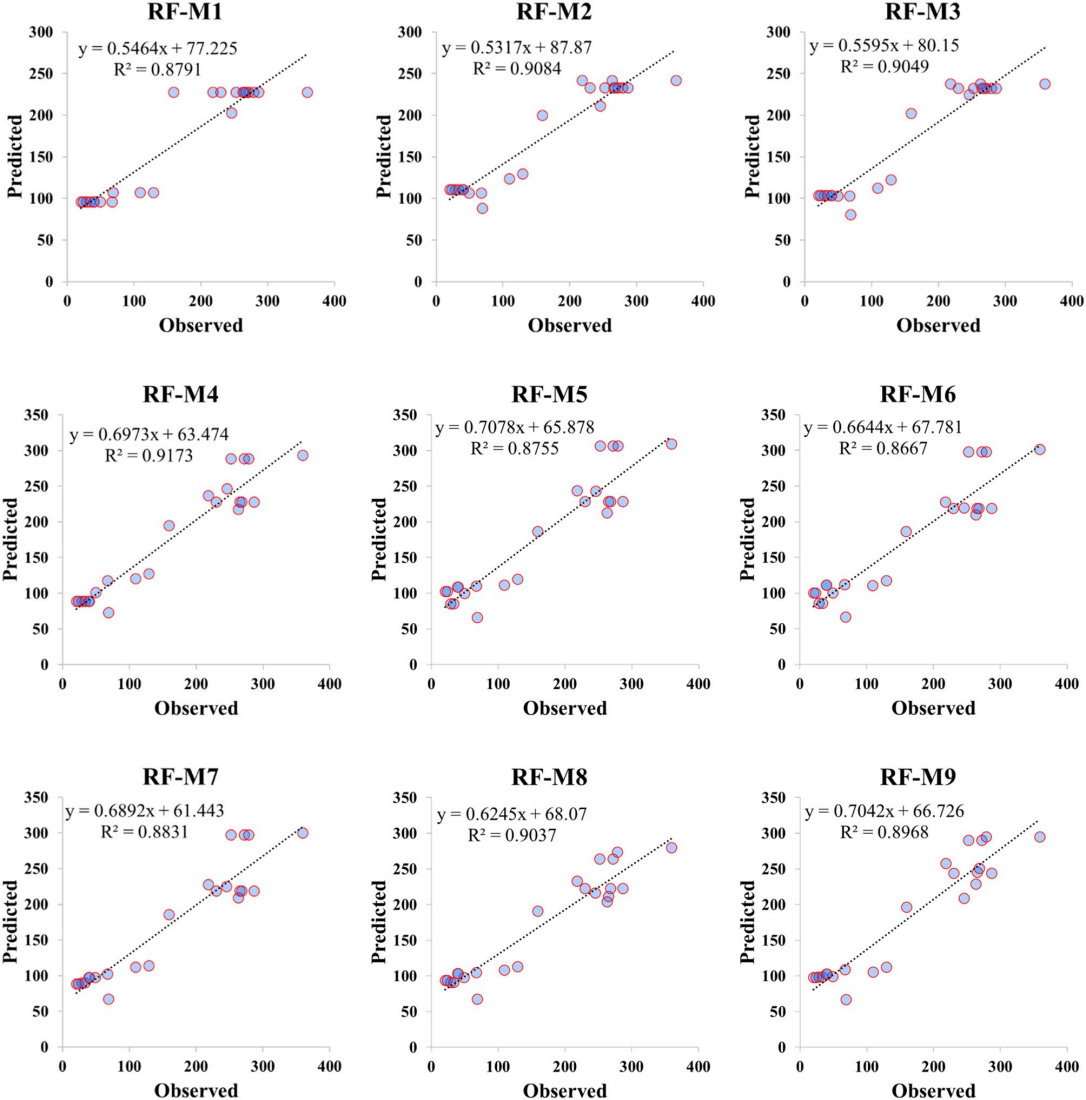
Scatter plot presentation of RF prediction model over the testing modeling phase.

Performance accuracy of the three ML models was also examined graphically by developing scatter plot, Taylor diagram and box plot. Figures 4, 5 and 6 demonstrated the scatter plot drawing for the applied models over testing phase which verified the linear relationship between the observed and predicted value of the *V*_*s*_. It can be recognized that M5 model presented an excellent fit with coefficient of determination more than 0.87 for all parameter combinations except M5 combination where statistical correlation reduces to 0.6484. ELM model showed a good predictability of *V*_*s*_ behavior over testing phase for all input combinations with R^2^ maxed out 0.85 except M1 combination where R^2^ has poor value and equal to 0.5388. RF model exhibited an excellent performance in *V*_*s*_ prediction when applied for both few and all predictor combinations. All statistical correlation values for RF model were ranged from 0.8667 to 0.9173 which revealed a good fit for *V*_*s*_ prediction.

**Figure 7:**
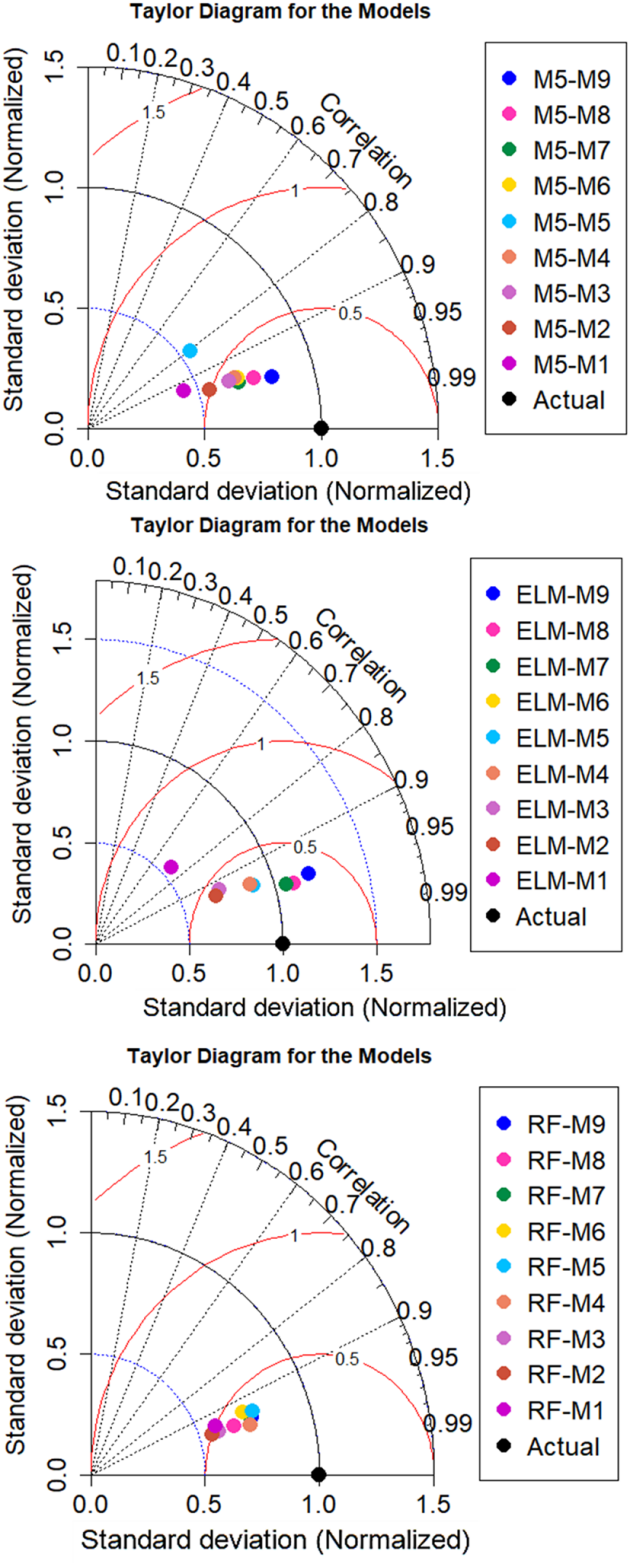
Taylor representation of ML models for testing phase.

Taylor diagram is constructed as a graphical representation to show the position of the developed algorithms with respect to the actual value based on three metrics including standard deviation, statistical correlation and RMSE^84^. Figure 7 depicts the Taylor representation of the three ML algorithms with all input combinations for testing phase. It can be noticed that the nearest distance to the actual value is obtained by using M5 model with nine parameters input parameters. The distance of the rest input combinations also attained high performance with regard to their distance to the actual point except M5 combination which has gained the less values of statistical correlation and standard deviation than the other combinations. Taylor graph for ELM model showed that applying eight input parameters proved the nearest performance to the actual value while the furthest point has gained by applying one input parameter. For RF model, all input combinations revealed good position to the actual *V*_*s*_ and the maximum values of correlation and standard deviation were achieved by applying four input parameters.

**Figure 8:**
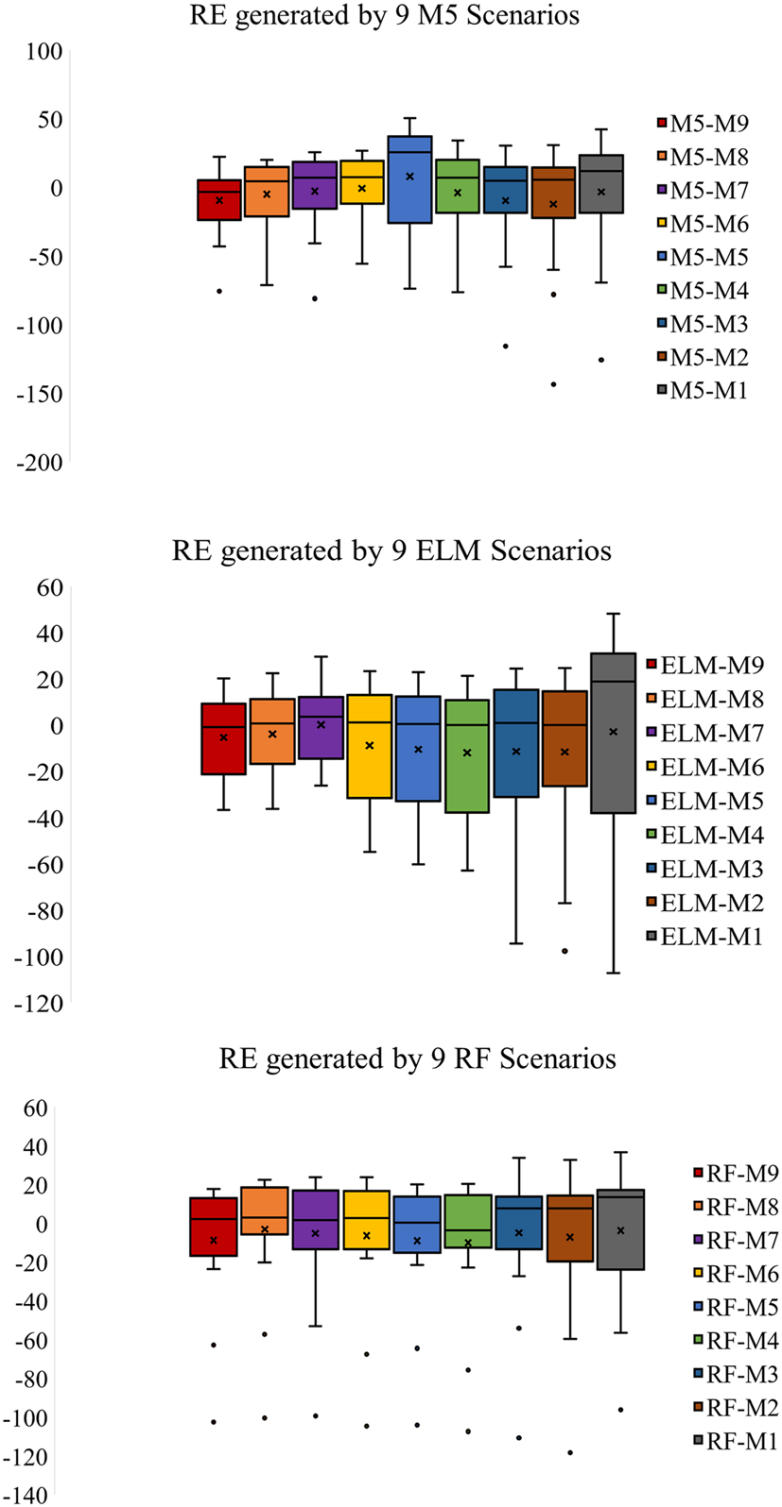
Residual error presentation of the applied ML models for testing phase.

Box plot presentation also generated in Fig. 8 to depict the relative error between observed and presented ML models for testing phase. M9 combination showed the less residual error than the other combinations of M5 models the negative outliers appeared in four input combinations including M4, M5, M6 and M8. For ELM model, the minimum error is gained by M7, M8 and M9 without negative outliers. The maximum error is demonstrated by ELM-M1 while ELM-M2 gained less error than M1, even though it has negative error outlier point. RF model combinations show that the least maximum error was achieved by M4, M5, M8 and M9 models while the fewest minimum error appeared in M4, M5 and M6 with error value less than 20%. Amon all RF constructed models, M4 and M8 combinations showed a reliable predictive performance with the least range value between first and third quartile and fewest maximum residual errors.

### Validation against the previous studies

To confirm the ability of constructed ML models in *V*_*s*_ prediction of FRP reinforced concrete, it is important to validate the presented models with the previously developed models over past studies. Nehdi et al.^85^ used genetic algorithm (GA) to propose *V*_*s*_ equations of FRP reinforced concrete beams using dataset with and without shear reinforcement. The results showed that the proposed model was an effective method in *V*_*s*_ prediction with R^2^ equal to 0.799. Oller et al.^86^ presented a mathematical equation for *V*_*s*_ prediction of FRP reinforced concrete beam with transverse reinforcement. The model applied using 112 samples and the *V*_*s*_ indicated good prediction performance with R^2^ = 0.862. Chou et al.^87^ used the hybrid model (i.e., SFA-LSSVR) for shear prediction using 209 samples of reinforced concrete beam with FRP reinforcement. The study showed that the presented model has reliable predictability with statistical correlation equal to 0.979. Recently, Alam et al.^45^ developed a hybrid model named BOA-SVR to predict the capacity of FRP reinforced concrete elements in *V*_*s*_ prediction. The model is tested based on 216 samples of FRP reinforced concrete with no transverse reinforcement and the results revealed that the developed models have high reliability in *V*_*s*_ prediction with R^2^ = 0.955. In the current study three advance ML methods including M5, ELM and RF model were tested in prediction process of shear capacity of 112 tests results of FRP reinforced concrete beam with transverse stirrups. None of the reported studies tested the impact of using different input parameter combinations in shear design modelling whereas in the current work a correlation statistic was used to construct nine combinations of input parameters and incorporated them with ML models in *V*_*s*_ prediction. All the developed models performed well in *V*_*s*_ prediction from one input to nine input parameters and the best prediction accuracy was exhibited by M5 model with nine input parameters.

### Discussion

Application of ML models in complex process such as *V*_*s*_ is highly needed to accurately simulate the nonlinear relationships between input and output parameters. The comparison analysis of the developed models revealed the reliability of the proposed methods because all algorithms achieved an excellent prediction performance except ELM-M1 and M5-M5 models which achieved R^2^ less than 0.70. Application of correlation methods in inputs construction showed that the beam width and reinforcing ratio of the transverse reinforcement are the most correlated parameters with *V*_*s*_ which revealed the importance of beam dimensions and stirrups in preparing shear design equation. M5 model showed a significant prediction accuracy when using few input parameters where RMSE equal to 57.4213 and 66.1925 for M5-M1 and M5-M2 respectively as shown in Table 5. Only M5-M5 model revealed poor reliability in shear estimation which has gained R^2^ = 0.6484 and high maximum residual error as depicted in Figs. 4 and 8. For ELM model, the least RMSE is attained by ELM-M8 and ELM-M7 with values equal to 33.832 and 34.4591 respectively as indicated in Table 5. Based on Taylor graph and box plot (see Figs. 7, 8), ELM-M1 showed the worst prediction accuracy with high negative error and the furthest position to the actual *V*_*s*_ which revealed the inability of ELM model to understand *V*_*s*_ behavior with only one input parameter. ELM requires more parameters to increase its performance for example ELM-M8 showed the nearest value to the observed value with high correlation and standard deviation as revealed in Fig. 7. With respect to RF model, the minimum RMSE is attained by using four input parameters with value equal to 42.7 (see Table 5). Both Taylor and box blot (see Figs. 7, 8) showed that RF model revealed a reliable predictability for all input combinations, even though it produced negative error outliers in all models. The performance analysis showed that the best coefficient of determination was gained by RF-M4 followed by RF-M2 as depicted by Fig. 6. Considering the performance results of the applied ML models, all models exhibited excellent results when input parameters were increased in modelling process. In the case of few input parameters, M5 and RF models perform better than ELM especially when they applied to one input parameter. The comparison analysis suggests that tree based model gained excellent results in capturing nonlinear relationship of *V*_*s*_ based on limited input parameters. The study revealed the ability of the proposed models in simulating the complex problem of shear behavior with a reliable and valid prediction. for future work, advance feature selection methods such as GA and extreme gradient boosting (XGBoost) can be introduced to capture nonlinear relationship between input and output parameters. These methods can be integrated with recent ML models such as deep learning algorithm to reduce residual error and perform more accurate results^54^.

## Conclusions

Development of a reliable and valid model in estimation shear behavior of concrete beam reinforced by FRP bars is an important step in the structural design concept. In the current research three popular ML models named M5 tree, ELM and RF model have been applied to estimate the shear capacity of FRP reinforced concrete beam with transverse reinforcement. Dataset including 112 shear samples were collected from previous works and a statistical correlation was conducted to construct input parameters combinations. Based on correlation value, a combination of nine input parameters were generated and used to test *V*_*s*_ of FRP reinforced concrete beam by the developed ML models. The presented algorithms were evaluated by using statistical validation and graphical methods. The statistical comparison showed that all generated models performed well for all input combinations except ELM-M1 and M5-M5 where their results are below the acceptable performance. The graphical evaluation revealed that the best results were attained by M5 tree with nine input parameters by scoring the highest coefficient of determination and minimum residual error. Furthermore, ELM and RF models showed their potential ability to enhance the predictive performance of shear behavior. All results demonstrated the ability of ML models in capturing the complex relationship of *V*_*s*_ in FRP reinforced concrete incorporating the impact of stirrups. For future study, GA and XGBoost should be explored to generate significant input selection. Also, deep learning model needs to be investigated to enhance the predictability of *V*_*s*_ performance. Finally, uncertainty analysis can be done to investigate the variability of the input parameters and the proposed models.

## Data Availability

Data can be provided upon request from the corresponding author.
